# Cultivating one health antibiotic stewards to bridge translational science gaps in the global action plan

**DOI:** 10.1016/j.onehlt.2022.100386

**Published:** 2022-04-05

**Authors:** Oladele A. Ogunseitan

**Affiliations:** a University of California, Irvine, Population Health & Disease Prevention, CA 92697, United states of America; b USAID One Health Workforce | Next Generation Project, United states of America; c NIH-funded Institute for Clinical and Translational Science, United states of America; d UC Global Health Institute, United states of America

**Keywords:** Antibiotic stewards, Pollution, Environment, Disease burden, Waste management, Sub-Saharan Africa, BAMR, Bacterial Antimicrobial Resistance, GAP, Global Action Plan, GLG, Global Leaders Group, SSA, sub-Sahara Africa, TIPH, This is Public Health

## Abstract

Scientific evidence for the urgency of curbing the emergence and spread of antimicrobial resistance is incontrovertible. Yet, the translation of knowledge into effective design and implementation of action plans is hampered by gaps in perception, attitudes and practices in the human health, agriculture, and environmental sectors. To fill these gaps in regions where the disease burden attributable to antimicrobial resistance is heaviest, a cadre of One Health Stewards equipped with strategies to translate and meld global and local evidence for knowledge dissemination is deemed necessary. This opinion articulates a case for cultivating and deploying One Health Antibiotic Stewards according to specific actions within the environmental context of antibiotic resistance.

## 1 Opinion

Scientific evidence for the urgent need to curb the emergence and spread of antimicrobial resistance is incontrovertible. Yet, the translation of knowledge into effective design and implementation of action plans is hampered by gaps in perception, attitudes and practices in the human health, agriculture, and environmental sectors. To fill the gaps in regions where the disease burden attributable to antimicrobial resistance is heaviest, a cadre of One Health Stewards equipped with strategies to translate and integrate global and local evidence for action-oriented knowledge dissemination is necessary. This opinion articulates a case for cultivating and deploying One Health Antibiotic Stewards according to specific actions within the local contexts in which antibiotic resistance manifests.

A comprehensive assessment of implementation of the global action plan (GAP) on antimicrobial resistance in the African region of the World Health Organization concluded that the overall implementation is inadequate, and that individual indicators showed a lack of progress, calling for urgent action [1]. The Global Leaders Group (GLG) on Antimicrobial Resistance also issued a call to protect the environment from antimicrobial pollution [2]. The urgency of the situation in sub-Sahara Africa (SSA) is supported by the 2019 assessment of the global burden of bacterial antimicrobial resistance (BMAR) which identified SSA as the region with the highest all-age death rate attributable to resistance at 27.3 deaths per 100,000 (20.9–35.3). The gap between the burden of BMAR in SSA and the region with the lowest burden, Australasia at 6.5 deaths (4.3–9.4) per 100,000, is more than four-fold [3]. The gap will likely widen if advances in translational science and implementation of antibiotics stewardship already underway in many regions of the world are not adopted for SSA, particularly in the western subregion of Africa, which was identified as carrying the heaviest burden of BMAR. The discrepancy between the heavy burden and the lofty expectations of the GLG supports the need for One Health Antibiotic Stewards for each of the four GAP pillars to strengthen weak links in the translation of knowledge and implementation of programs for reducing the burden of antibiotic-resistant infections.

The GLG, established late in 2020 just before the COVID-19 pandemic, aimed to stimulate and sustain collaboration across governments, agencies, civil society and the private sector through a One Health approach, and its action may be key in disseminating best practices and advocating for global resources for implementation of effective interventions in resource poor regions. It is doubtful, however, that without grassroot community engagement in strategies to combat BAMR, top-level interventions in the GLG call-to-action for reducing antimicrobial discharges from food systems, manufacturing facilities and human health systems into the environment will be effective for sustained impact. The following sections highlight potential roles for national or community-level Antibiotic Stewards in each of the four pillars identified in the GLG call-to-action.

## 2 Governance and oversight

The first pillar regards the call to strengthen governance and oversight, the implementation of regulatory frameworks, and standard operating procedures to establish safe levels, better control and monitor the distribution and release of antimicrobials, antimicrobial resistant bacteria and antimicrobial resistance determinants from food systems, manufacturing facilities and human health systems into the environment. In many SSA countries, controlling the use of antibiotics in food systems is likely dominated by agricultural feedstock with a limitation that information about active ingredients is generally not available through bulk retailers, and the comprehension of available information to support decision-making by farmers and animal herders is not assured. Thus, bridging the gap between government policies and farm-level implementation demands an occupation-focused information campaign with Antibiotic Stewards that stresses the One Health communication strategy. Governance and oversight also involve development and implementation of stewardship policies and protocols for responsible and sustainable use and procurement of antimicrobials and the enforcement of regulations to reduce or eliminate inappropriate use that is not supported by credentialed health care provider or veterinarian. This would require large-scale behavior modification and management of expectations by consumers and retailers if the strategy is to prevent inequity in access to medications.

## 3 Surveillance and data availability

The second pillar, to improve surveillance and data availability specifically to fill gaps in knowledge about the fate, concentration and impact of antimicrobial contamination and pathogen discharges on the environment cannot be meaningfully implemented without building the necessary workforce and technical staff in biochemistry, microbial ecology, and field epidemiology. Recruitment of Antibiotic Stewards open to short term training for field sampling could be a way to empower citizen scientists covering a broad geographic range. Such programs will require establishment of sample processing centers and a cold-chain sample transportation infrastructure to guarantee data quality. Circumvention of these infrastructure difficulties in SSA would require development of rapid response field diagnostic tool s for detecting and measuring antibiotic residues in the environment, and for molecular-level identification of bacterial strains, particularly among the six pathogens which account for the highest number of deaths attributable to BAMR, namely, *Escherichia coli*, followed by *Staphylococcus aureus, Klebsiella pneumoniae, Streptococcus pneumoniae, Acinetobacter baumannii,* and *Pseudomonas aeruginosa*.

## 4 Water, sanitation and hygiene

The third pillar, to improve discharge management, emphasizes water, sanitation and hygiene (WASH), vaccination, biosecurity and animal husbandry. At any given period of time, there are numerous WASH projects being implemented in SSA, but it is difficult to assess the sustained impact on BMAR because this topic has not been the traditional focus of such projects. Access to disinfected water systems is a major limitation in this regard, and government programs need to be supplemented with information campaign that could be delivered through broad recruitment and education of Antibiotic Stewards.

## 5 Research and development

Finally, the fourth pillar addresses research and development, including improved understanding and deployment of greener waste management technologies and procedures for degrading antimicrobial chemical residues, resistance genes and resistant organisms in waste disposal facilities. Unfortunately, landfill disposal and open burning of domestic waste are prevalent in SSA, and in the absence of more centralized waste management infrastructure, these systems will remain vulnerable as hot spots of BAMR breeding and dissemination. However, public education about the disposal of medical waste and specially-designated collection sites may be within reach of most communities, and Antibiotic Stewards may catalyze action through establishment and maintenance of communities of practice. Stimulating research also require government funding, and in this regard, training in the writing of policy briefs on antimicrobial resistance and in strategies for communicating with policymakers across the ministries of health, environment, agriculture, and education is consistent with the One Health approach, and will help cultivate support for increasing research funds allocation and evidence-based policymaking to curb the adverse impacts of BMAR. There is credible evidence that the spread of BMAR can be exacerbated by climate change, a topic that is currently very salient, but underfunded in terms of research on vulnerable populations. Leveraging the salience of topics such as climate change to support research on the high burden of BMAR could be presented as a win-win program.

## 6 Empowering one health antibiotic stewards

Clearly, BMAR is an emergency jeopardizing population health, food security, and environmental quality. Proposed remediation strategies hinge on improving antibiotic stewardship through deeper knowledge, conservative attitudes, and preventive practices across agriculture, environmental, and human health sectors, demanding a quintessential One Health approach. An example of how to proceed with cultivating and empowering local One Health Antibiotic Stewards is pertinent. In 2021, the Global Network for Academic Public Health, consisting of Regional Association Members Alianza Latinoamericana de Salud Global (ALASAG); Asia-Pacific Academic Consortium for Public Health (APACPH); Association of Schools of Public Health in Africa (ASPHA); Association of Schools of Public Health in the European Region (ASPHER); Association of Schools and Programs of Public Health (ASPPH); South East Asia Public Health Education Institutions Network (SEAPHEIN) launched our project through a “This is Public Health - Global” (TIPH-Global) initiative. The aim is to translate local knowledge into global action against the growing threat of antibiotic-resistant infections. This aim requires improved calibration of the wide range of public knowledge, attitudes and practices, not only internationally, but within various population and occupational constituencies at the national level. For example, through a cross-sectional study that we conducted in twelve communities in the urban Greater Accra and rural Upper West regions of Ghana, we showed that antibiotic knowledge, attitudes, and use varied significantly across demographics, which suggests the need for context-specific development of effective community interventions [4]. The specific intervention of the TIPH-Global initiative, entitled “Antibiotic Stewardship is Public Health” is to use data gathered at the community-level for designing an information dissemination campaign through the recruitment of “Antibiotic Stewards”.

In a pilot study to recruit such One Health Antibiotic Stewards, we noted the dearth of knowledge about the environmental context of BMAR at the individual level, and the lack of infrastructure at the community level including, for example, facilities to collect expired antibiotics for proper disposal. Recruited Antibiotic Stewards are expected to wear apparel printed with a specially designed logo to stimulate public conversation and information dissemination to expand the reach of the program [5]. In many SSA countries where certain antibiotics may be purchased from neighborhood stores, and domestic and agricultural sewage and solid waste treatment are not centralized, the possibility and frequency of emergence of BMAR is expected to be higher than in regions with broader and deeper investments in environmental stewardship [6].

## Declaration of Competing Interest

The author declares that there is not conflict of interest.
